# Multi-level strategies to tailor patient-centred care for women: qualitative interviews with clinicians

**DOI:** 10.1186/s12913-020-05082-z

**Published:** 2020-03-14

**Authors:** Tali Filler, Sheila Dunn, Sherry L. Grace, Sharon E. Straus, Donna E. Stewart, Anna R. Gagliardi

**Affiliations:** 1 Toronto General Hospital Research Institute, University Health Network, 200 Elizabeth Street, Toronto, Ontario M5G2C4 Canada; 2 Women’s College Hospital, Toronto, Canada; 3 York University, Toronto, Canada; 4 St. Michael’s Hospital, Toronto, Canada

**Keywords:** Patient-centered care, women’s health, Gender issues, Professional-patient relations, Quality of health care, Attitude of health personnel, Qualitative interviews

## Abstract

**Background:**

Patient-centered care (PCC) is one approach for ameliorating persistent gendered disparities in health care quality, yet no prior research has studied how to achieve patient-centred care for women (PCCW). The purpose of this study was to explore how clinicians deliver PCCW, challenges they face, and the strategies they suggest are needed to support PCCW.

**Methods:**

We conducted semi-structured qualitative interviews (25–60 min) with clinicians. Thirty-seven clinicians representing 7 specialties (family physicians, cardiologists, cardiac surgeons, obstetricians/gynecologist, psychiatrists, nurses, social workers) who manage depression (*n* = 16), cardiovascular disease (*n* = 11) and contraceptive counseling (*n* = 10), conditions that affect women across the lifespan. We used constant comparative analysis to inductively analyze transcripts, mapped themes to a 6-domain PCC conceptual framework to interpret findings, and complied with qualitative research reporting standards.

**Results:**

Clinicians said that women don’t always communicate their health concerns and physicians sometimes disregard women’s health concerns, warranting unique PCC approaches.. Clinicians described 39 approaches they used to tailor PCC for women across 6 PCC domains: foster a healing relationship, exchange information, address emotions/concerns, manage uncertainty, make decisions, and enable self-management. Additional conditions that facilitated PCCW were: privacy, access to female clinicians, accommodating children through onsite facilities, and flexible appointment formats and schedules. Clinicians suggested 7 strategies needed to address barriers of PCCW they identified at the: patient-level (online appointments, transport to health services, use of patient partners to plan and/or deliver services), clinician-level (medical training and continuing professional development in PCC and women’s health), and system-level (funding models for longer appointment times, multidisciplinary teamwork to address all PCC domains).

**Conclusions:**

Our research revealed numerous strategies that clinicians can use to optimize PCCW, and health care managers and policy-makers can use to support PCCW through programs and policies. Identified strategies addressed all domains of an established PCC conceptual framework. Future research should evaluate the implementation and impact of these strategies on relevant outcomes such as perceived PCC among women and associated clinical outcomes to prepare for broad scale-up.

## Background

Patient-centered care (PCC) is an essential component of high-quality health care. Initially recognized by the Institute of Medicine in 2001, PCC engages patients and families so that care is tailored to clinical needs, life circumstances and personal preferences [1]. Since then, our understanding of PCC has evolved to a multidimensional approach whereby clinicians foster a healing relationship, exchange information, respond to emotions, manage uncertainty, engage patients in decisions, and enable self-management [2]. Considerable evidence demonstrates that PCC enhances the patient experience and numerous clinical outcomes [3, 4]. Hence, it is widely advocated by many organizations. For example, in 2018 the American College of Physicians published a position paper including four principles for authentic PCC and advice on implementing PCC in practice [5]. On a broader scale, the World Health Organization published a global strategy on people-centred health services comprised of policy options and interventions organized by strategic objectives aligned with five goals: empower and engage people, strengthen governance and accountability, reorient the model of care, coordinate services, and create an enabling environment [6].

Despite its known benefits [3, 4] and advocacy by professional organizations [5, 6], research shows that many clinicians face barriers in implementing PCC. Barriers reported by American geriatricians included comfort with the traditional role of principal decision-maker, lack of incentives and payment structures, lack of guidance, and medico-legal concerns when patients make decisions with which physicians strongly disagree [7]. Through interviews, 86 Jordanian surgeons and oncologists said that insufficient time to spend with patients, patient indecision, and family members overriding the decision-making process were barriers of PCC [8].

As a result of such barriers, many patients do not experience PCC and its associated benefits. An American population-based survey of 2718 adults who saw a physician for medication for hypertension, elevated cholesterol, or depression; screening for breast, prostate, or colon cancer; knee or hip replacement for osteoarthritis, or surgery for cataract or low back pain revealed variable involvement in discussion and decision-making, important PCC components [9]. A Commonwealth Fund survey revealed that, among high health care users, women were less likely than men to have medical needs addressed, access to a specialist, or report good patient-provider communication [10]. A cross-sectional survey of 3625 women in Kenya, Ghana or India regarding patient-centred maternity care found that a high proportion of physicians never introduced themselves (90%), asked permission before performing medical procedures (57%), or explained the purpose of procedures (69%) or prescribed medications (58%) [11]. Gendered inequities in health care quality are a persistent health care problem with negative individual and societal implications [12].

PCC is one approach for ameliorating gendered inequities in health care quality, and the basis for a 2018 United Nations Report, Gender Equality in the 2030 Agenda for Sustainable Development [13]. In a prior scoping review, we reported that no studies investigated how clinicians tailor patient-centred care specifically for women (PCCW) [14]. Hence, knowledge of what constitutes and challenges PCCW is needed to inform the development of strategies or interventions that help clinicians address women’s needs and preferences. The purpose of this study was to explore how clinicians achieve PCCW, the challenges they face in doing so, and the strategies they suggest are needed to support PCCW.

## Methods

### Approach

Given limited research on clinician perspectives of PCCW, we employed a qualitative research design [15]. Specifically, we used a descriptive qualitative approach involving semi-structured interviews [16]. This method aims to capture straightforward accounts of participants’ views and experiences, and does not generate or test theory [17]. To optimize rigour, we sampled participants with various characteristics that may influence PCCW views and experiences, used inductive analysis so that findings emerged from the data, involved multiple researchers in independent analysis to enhance reliability, and consulted with stakeholders as a form of member checking [18]. Stakeholders included the research team, comprised of health services researchers, physicians of various specialties, experts in women’s health and/or PCC, representatives of professional societies, charitable foundations, quality improvement agencies, and patient research partners, who provided input at all stages. To further ensure rigour, we complied with the 32-item Consolidated Criteria for Reporting Qualitative Research (COREQ) [19]. This study was approved by the University Health Network Research Ethics Board. All participants were informed about the study’s purpose and provided written informed consent prior to interviews. There was no prior relationship between the interviewer and participants.

### Sampling and recruitment

We used purposive sampling to recruit clinicians who varied by characteristics that could influence their PCCW views or experiences including specialty (family physicians, cardiologists, cardiac surgeons, obstetricians/gynecologist, psychiatrists, nurses, social workers), region (across the province of Ontario, Canada), setting (urban, rural), self-reported years in practice (early, mid, late), and gender (female, male). We chose specialities who could speak about one of three health care issues. These included care for cardiovascular disease and depression, conditions with known gendered inequities [20, 21]. We also sampled clinicians who could speak about contraceptive counseling because it is a common issue among women, who value interpersonal communication and engagement in decision-making, factors associated with contraceptive use, yet are frequently dissatisfied with counseling [22, 23]. We identified physicians using the publically available College of Physicians and Surgeons of Ontario directory. They were invited to participate via mail, email, phone and fax. Interviewed physicians referred us to nurses and social workers (snowball sampling). We aimed to interview 10 clinicians for each of contraceptive counseling, cardiovascular disease and depression who varied in non-mutually exclusive fashion for other sampling characteristics. Sampling was concurrent with data collection and analysis, and proceeded until data saturation, when no new themes emerged from further interviews, as established through research team discussion.

### Data collection

Telephone interviews were conducted between July 2018 and January 2019 by MSc-trained research associates DK and BN, both with qualitative research experience. They were mentored by ARG, a PhD-trained health services researcher with considerable experience in qualitative research. The semi-structured interview guide developed for this study included three questions. To explore how clinicians delivered PCCW, we asked: *How do you tailor PCC for women?* We also asked: *What factors challenge PCC for women?*, and W*hat strategies or interventions would help you deliver or achieve PCC for women?* DK, BN and ARG independently analyzed the first four transcripts, and discussed and agreed upon revisions to improve wording and flow of interview questions. The research team also reviewed preliminary data and the interview guide, and offered additional suggestions to improve wording of questions. Interviews ranging from 25 to 60 min were audio-recorded and transcribed verbatim by an external professional.

### Data analysis

We identified themes inductively using constant comparative technique and used Microsoft Office (Word, Excel) to manage data [24]. BN, DK and ARG independently coded the first four transcripts, and compared and discussed coding to develop a preliminary codebook of themes and exemplar quotes (first level coding). DK and MSc-trained research associate TF, also with qualitative research experience, then similarly analyzed remaining transcripts. TF, with periodic guidance from ARG, expanded or merged themes (second level coding). To assist in interpreting findings, identified themes were then mapped to six PCC domains (foster a healing relationship, exchange information, address emotions/concerns, manage uncertainty, make decisions, enable self-management) based on a conceptual framework developed by McCormack et al. [2], chosen from among others because it was comprehensive and rigorously-developed [25]. Themes and exemplar quotes were shared with the research team on October 16, 2018, and their feedback was used to refine themes. Summary data were shared with 17 women and 24 clinicians at a one-day meeting on February 27, 2019 as an additional form of member checking to enhance clarity of the findings. We reported themes and select exemplar quotes, noting whether most, several or few participants articulated themes, and summarized additional exemplar quotes in Additional File 1.

## Results

### Participants

Of 369 clinicians contacted, 327 did not respond, 42 consented, and of those, 37 scheduled an interview to discuss PCCW for one health care issue: depression (*n* = 16), cardiovascular disease (*n* = 11) or contraceptive counseling (*n* = 10) (Table 1).

**Table 1:** Participating clinician characteristics

Characteristic	n (% of 37)
Specialty	
Psychiatrist	8 (21.6)
Cardiologist	6 (16.2)
Family Physician	11 (29.7)
Obstetrician and Gynecologist	1 (2.7)
Cardiac Surgeon	1 (2.7)
Social Worker	1 (2.7)
Registered Nurse	9 (24.3)
Health care issue	
Cardiovascular disease	11 (29.7)
Depression	16 (43.2)
Contraception counseling	10 (27.0)
Setting	
Urban	25 (67.6)
Rural	12 (32.4)
Self-reported years of practice	
< 10 years	12 (32.4)
>/= 10 years	25 (67.6)
Gender	
Women	28 (75.7)
Men	9 (24.3)

### Women have unique PCC needs

Clinicians emphasized that women face unique challenges in seeking health care and communicating about health care issues that warrant tailoring of PCC. They perceived that *women were reluctant to speak up about concerns* due to shame, fear of judgment, age, culture, negative prior health care experience, or prioritizing family over their own health.

*They have these feelings, they view it as a weakness, it’s something shameful, they don’t see it as something that they need treatment for (001 man clinician, depression, rural).*

*Particularly if we’re talking about speaking with very young women, or women from certain cultural or religious backgrounds, or someone who has had sexual trauma or negative experiences with the health care system (032 woman clinician, contraception, urban).*

*Women have more challenges than men because women tend to be the caregivers within a relationship for older people and for their family in the younger age group. It’s harder for a woman to have an operation than it is for a man (005 male clinician, cardiovascular, urban).*

Clinicians also perceived that *concerns articulated by women are dismissed or overlooked*, and as a result they do not access needed health care services.

*In general, women aren’t taken as serious … physicians don’t listen to women in quite the same way they do men (020 woman clinician, cardiovascular, urban).*

*One of the problems in our system is that women complain that they are not heard (008 man clinician depression urban).*

*I just talked to a female patient two weeks ago who said that she had presented three times to the family doctor with symptoms and the family physician deemed it a panic attack but she pushed back and was eventually referred to cardiology and had since had [coronary artery bypass grafting] (011 woman clinician, cardiovascular, urban).*

### Tailoring PCC for women

Themes and exemplar quotes are summarized in Additional File 1, and themes are discussed here with select exemplar quotes. Clinicians described 39 approaches they used to tailor PCC for women across 6 PCC domains (Table 2). There was little discrepancy in themes across health care issue or clinician characteristics, suggesting a broadly-relevant set of behaviours to deliver or achieve PCCW.

**Table 2:** Strategies to tailor patient-centred care for women

PCC domain	Themes and corresponding strategies
Foster a healing relationship	Establish rapport • Engage in brief, friendly discussion prior to clinical discussion • Ask patients to share some information about themselves • Share some information about yourself to find common Build trust • Listen to the patient • Adopt non-judgmental facial expression/tone of voice • Make eye contact by facing the patient (not a computer) • Sit in a relaxed manner across or beside the patient
Exchange information	Explore patient context • Set aside clinical agenda/allocate time to explore patient context • Identify contextual factors such as age, lifestyle, culture or race, and socioeconomic status • Discuss patient values and goals/revisit over time Assess and support patient knowledge • Ask patients what they understand about their health care issue, condition or treatment • Use lay language • Employ visual aids to supplement discussion • Ask patients to summarize details in their own words
Address emotions or concerns	Elicit emotions or concerns • Ask if patient has emotions, concerns, worries or discomfort • Allocate time to discuss those issues Validate emotions or concerns • Assure patient that what they feel is normal and common • Provide enough time for patient to fully express themselves • Allow the patient to take breaks if needed or revisit the topic at a later time Provide or refer to supportive resources • Provide advice or informational material about issues causing emotions or concerns, or to help patient manage them • Refer women to resources: informational material, support groups, social worker, etc.
Manage uncertainty	Acknowledge uncertainties • Explicitly mention uncertainties about prognosis, and the risks and benefits of treatment options • Discuss uncertainties related to patient’s contextual factors (i.e. age, lifestyle, health status) Provide educational material • To supplement discussion, provide educational material to help patients understand the nature, risk and impact of uncertainties
Make decisions	Collaborate on decisions • Describe available management options • Ask patients about preferences given their contextual factors • Allow patient to make final choice; otherwise, they may not comply Involve family members or care partners • If desired by the patient, extend collaboration to family members or care partners • Invite them to ask questions by telephone • Involve them in appointments
Enable self-management	Offer flexible follow-up options • In addition to traditional in-person visits, offer home-based follow-up through telephone or computer Tailor self-care plans • Jointly plan self-management strategies with patients to accommodate preferences and contextual factors • Provide instructions and informational material to support self-care including how to self-monitor health and what to do if symptoms change or worsen • Refer patients to informational material or support groups
Additional conditions	Ensure privacy so that women feel safe and comfortable sharing information • Offer women-only hours or services • Maintain a separate waiting area or clinic space for women • Provide access to female clinicians Accommodate children so that women can seek care/manage their own health • Allow women to bring children to appointments • Offer a play area or child care • Maintain flexible appointment schedules (evening/weekend) for women who must remain at home with children during the day

#### Foster a healing relationship

Most clinicians explained that they employed two approaches for fostering a healing relationship: *establish rapport* and *build trust*. They established rapport by engaging in brief, friendly conversation prior to discussion of a clinical nature, and by asking women to share some information about themselves and/or sharing personal information with patients to find common ground. Clinicians said they built trust by listening to the patient, adopting a non-judgmental tone of voice and facial expression, asking for permission to touch them, and through body language, meaning facing the patient to make eye contact rather than facing a computer screen, and sitting in a relaxed manner either across from or beside patients.

I try to find something that is common … and try to initially make it a little bit lighter so to make them relaxed (05 male, cardiovascular disease, urban).

#### Exchange information

Most clinicians described two approaches for exchanging information: *explore patient context* and *assess and support patient knowledge*. They explored context, meaning patient goals and priorities, by temporarily setting aside their own clinical agenda and allocating time to determine what is important to the patient, and revisiting patient goals over time. Other contextual details they gathered to learn about patient context included age, lifestyle, culture or race, and socioeconomic status. Clinicians said they assessed knowledge by asking patients what they understood about their condition or treatment. To support knowledge, they tried to use lay language, employed visual aids such as charts or posters to supplement discussion, and asked patients to summarize discussion in their own words as a way of ensuring they understood key concepts.

Putting aside our agenda to some extent, of health care providers and, really trying to get a sense of what is important for the person sitting in front of you (32 female, contraception, urban).

### Address emotions/concerns

Several clinicians described three approaches to address emotions/concerns: *actively elicit emotions/concerns*, *validate emotions/concerns*, and *provide or refer to supportive resources*. They asked patients if they had questions or felt emotions, concerns, worries or discomfort, and allocated time to discuss those issues. Clinicians emphasized the need to validate emotions/concerns to assure patients that what they are feeling is common and distressing. They also provided patients with enough time to fully express themselves, and allowed them to take breaks when needed or schedule repeat visits. Clinicians also provided advice or informational material to address emotions, or referred women to resources such as organizations that offer informational material, support groups, or social workers.

reassuring them that it’s reasonable to feel the way they’re feeling and also that they’re not alone in feeling the way they’re feeling (01 male, depression, rural).

#### Manage uncertainty

A few clinicians described two approaches they used to manage uncertainty about prognosis and the risks and benefits of treatment options: *acknowledge uncertainties* and *provide educational material*. Clinicians said they explicitly mentioned and described uncertainties, and also relate uncertainties to aforementioned contextual factors such as age, lifestyle and health status. They also offered educational material or links to web sites to help patients understand the nature, risk and impact of uncertainties. This was done to equip patients for involvement in decision-making and to help support ongoing self-management.

I give them, if needed, reading materials or pamphlets … on medication side effects … they may meet with the pharmacist and talk about risks, benefits of treatment (15 female, depression, urban).

#### Make decisions

Several clinicians described two approaches for making decisions in a patient-centred way: *collaborate on decisions* and *involve family members/care partners*. Clinicians said that they employed shared decision-making, meaning they described available management options but asked patients about their preferences given various contextual factors. If patients were not involved in the final decision, clinicians perceived that they may not adopt and benefit from a given treatment. Clinicians also extended collaboration to family members or care partners if desired by the patient as a means of strengthening decision-making.

it’s only going to work if the goals of your treatment align with the goals of what the patient has in mind for their lifestyle and their priorities (10 female, cardiovascular disease, urban).

#### Enable self-management

Several clinicians noted two approaches for enabling self-management: *flexible follow-up options* and *personalized self-care plans*. Participants said that offering flexible options for follow-up appointments was critical to monitoring response to treatment and health status. Apart from traditional in-person consultations, follow-up options included the use of phone or computers for home-based appointments. Personalized self-care support referred to jointly planning self-management strategies with patients to consider preferences given contextual factors such as lifestyle. Clinicians also said they supported self-care by providing instructions and informational material on self-management strategies, how to self-monitor health, and what to do if symptoms change or worsen, or offering patients informational material, or links to web sites or support groups.

we’re growing home-based, computer-based and tele-rehab sites (10 female, cardiovascular disease, urban).

#### Additional conditions

Clinicians identified two additional themes pertaining to conditions that support PCCW: *ensure privacy* and *accommodate children*. Clinicians said that many women value a private environment, potentially through women-only hours or separate waiting and clinic areas, so that they feel safe, secure, and able to share information. Clinicians also emphasized that women are often more comfortable about sharing personal or intimate details with a woman rather than a man clinician. To help women manage their own health by accommodating children, participants said that it was important to allow women to bring children to appointments, offer a play area or child care, and maintain flexible appointment schedules such as evenings or weekends for women who must remain at home with small children during the day.

### Strategies needed to overcome barriers of PCC for women

#### Clinicians recognized that patients in general often lack PCC

*It’s always a struggle to be patient-centred because there’s always so many conflicting demands (016 man clinician depression urban).*

*Unfortunately, my experience in medicine has been that very frequently we don’t provide as good patient-centred care as I think would be beneficial. It tends to be very focused on algorithms and what we believe is going to work, and sometimes forgetting what it is that the patient actually wants and not even asking them sometimes (034, woman clinician, depression, rural).*

Clinicians identified barriers of PCCW at the patient, clinician and system level, and 7 corresponding strategies needed to address barriers (Table 3). Barriers at the patient level referred to contextual factors that influenced women’s health seeking or health behaviour. Potential strategies to overcome those barriers included online appointments or transport to health services for women not able to access services specialized in women’s health or those with childcare restrictions; and use of patient partners either to help plan services that are sensitive to women’s needs, or to assist in discussions or counseling at the patient level. At the clinician level, participants recognized they lacked knowledge or skill in PCC and/or women’s health, and recommended that medical training and continuing professional development address those topics.

*One thing we could do more is incorporate and teach the principles of patient-centred care into every day teaching experiences with our residents (021 man clinician contraception urban).*

*We need to incorporate it into the curriculum for internal medicine and cardiology trainees and in primary care as well (035 woman clinician cardiovascular urban).*

**Table 3:** Strategies needed to overcome barriers of patient-centred care for women

Level	Barriers	Strategies
Patient	Contextual factors (i.e. social determinants, culture) influence women’s health seeking and self-care behavior	• Online appointments or transport to health services for women otherwise unable to get there • Use of patient partners to help plan services sensitive to women’s needs, and assist in discussions, counseling, etc. at the patient level
Clinician	Lacking knowledge or skill in patient-centred care	Medical training and continuing professional development in PCC and in women’s health
Health System	Limited time during appointments to address all domains of patient-centred care	• Funding models that accommodate longer, more complex appointments • Multidisciplinary teams • Counseling, education and decision support by Physician Assistants or Nurse Practitioners
	Lack of resources to support patient-centred care	Funding and resources for clinics and services that specialize in or accommodate PCC for women

At the system level, participants said that funding was needed to address the lack of resources for clinics and services that specialize in or accommodate PCCW. In particular, most participants emphasized the challenge of delivering or achieving PCC given short appointment times. To overcome this limitation, they recommended funding models that accommodated longer appointment times, the use of multidisciplinary teams to deliver care in a way that addressed complex health needs, and the use of Physicians Assistants, Nurse Practitioners, Nurses and Social Workers to assume responsibility for counseling, education and decision support.

*Time is always a barrier. Sometimes we have quite a few patients and it’s difficult to spend a lot of time with all of them. Ideally we’d have more staffing but that’s very difficult in terms of finances (017, man clinician, cardiovascular, urban).*

*We can’t call ten minute appointments patient-centered care, they’re not. You can be the nicest doctor and make the most eye contact you want to, but sometimes ten minutes is just not enough (032 female, contraception, urban).*

## Discussion

This study explored how 37 clinicians with varied characteristics and representing 7 specialties who manage contraceptive counseling, cardiovascular disease or depression delivered or achieved PCCW. Clinicians identified 39 strategies across 6 PCC domains to support PCCW that could be broadly emulated by clinicians. Clinicians also identified 7 additional strategies that could be applied by health care managers or policy-makers to overcome patient-, clinician- and system-level barriers of PCCW. Themes were similar regardless of health care issue or clinician characteristics.

A large proportion of prior research that conceptualized PCC, including reviews of published research [26], and qualitative interviews [27], failed to separately report and therefore distinguish clinician and patient perspectives. Interviews with 107 staff, front-line clinicians, middle managers, and executives at 4 Veteran Health Administration medical centres applying patient-centred care transformation revealed that PCC was regarded as an overarching nebulous concept that encompassed everything and was already part of existing practice, thus providing little insight on how to implement PCC [28]. A review of interventions to achieve patient- and family-centred care, including 28 reviews published from 2011 to 2017, identified the need for educational interventions targeted at patients, family members, or providers to foster PCC [29]. This accumulated research did not identify which interventions were tested in, or suitable for patients with different characteristics, in particular, women. Little research has explored clinician views on PCC for women. In interviews, 16 American physicians and midwives said that patient-centred maternity care involved individualized care, two-way transfer of information, and actively engaging patients [30]. In contrast, our study identified many more strategies to implement PCC, and we analyzed findings according to an established framework that serves as an ideal by which to model PCC [2]. Thus, our research is unique in that it revealed numerous replicable strategies for implementing PCC specifically for women.

Applying these strategies to implement PCCW may not be easy or possible. In part, clinicians are constantly faced with the need to adopt new practices, pressure that can adversely affect job satisfaction and clinical performance [31]. Moreover, our research also identified multi-level barriers of PCCW, not all of which are actionable by clinicians. Clinicians acknowledged that they lacked knowledge or skill in PCC and women’s health, and recommended the need for medical training and continuing professional development in these areas. First, research may be needed to examine the content of medical curriculum to describe if and how PCCW is currently addressed, and if not, reveal how medical curriculum could be enhanced to equip future clinicians with knowledge and skill to support PCCW. Another key barrier identified in this study and in prior research was lack of time during appointments to address all PCC domains [7, 8]. Suggested strategies included multidisciplinary teams to share responsibilities that may include counseling, education and decision support, and clinician funding models that accommodate longer appointments. Considerable research demonstrates the positive impact of inter-professional teamwork on team behaviour, competencies, performance and clinical outcomes, and identifies approaches to support it including role-sharing and funding models [32, 33]. Such knowledge could inform the development and implementation of models by health care managers or policy-makers can facilitate teamwork for PCCW.

This study featured several strengths. We employed rigorous qualitative methods that complied with reporting standards [18, 19]. We included clinicians with a wide variety of characteristics, which further strengthens the relevance and validity of the findings. We also involved our multi-disciplinary research team that included researchers, clinicians and patient research partners in planning and undertaking data collection, analysis and interpretation; and reviewed findings with stakeholders including women patients at a one-day meeting to enhance clarity. A few limitations should also be noted. Participants were volunteers, thus their views about PCCW may reflect their interest in this subject, and may differ from the clinician populations they represent. Also, the findings may not be generalizable to clinicians in countries outside of Canada with differing contexts such as culture or health system. While many of the strategies may appear to also be relevant for men patients, they were identified by clinicians who were asked about tailoring PCC specifically for women; thus, further research would be needed to establish approaches that tailor PCC for men. Clinician views about PCCW may not match those of women; we also interviewed women about strategies to achieve PCCW (not yet published).

## Conclusions

This study generated practical and actionable insight that can help clinicians to tailor the implementation of PCCW, and health care managers and policy-makers to support PCCW implementation by clinicians. Future research should validate these strategies among larger groups of clinicians in different sites, then evaluate the implementation and impact of prioritized strategies on relevant outcomes such as perceived PCC among women and associated clinical outcomes.

## Supplementary information

**Additional File 1.** Themes and exemplar quotes for strategies to tailor patient-centred care for women. Table listing themes and exemplar quotes organized by domains of patient-centred care.

## Data Availability

The datasets used and/or analysed during the current study available from the corresponding author on reasonable request.

## Abbreviations

PCC: Patient-centred care
PCCW: Patient-centred care for women
