# Supplementary material for: Multi-level strategies to tailor patient-centred care for women: qualitative interviews with clinicians
Source: BMC Health Serv Res. 2020 Mar 14;20:212. doi: 10.1186/s12913-020-05082-z (PMC7071699; doi:10.1186/s12913-020-05082-z)
Supplement: Supplementary file 1 — Additional File 1. Themes and exemplar quotes for strategies to tailor patient-centred care for women. Table listing themes and exemplar quotes organized by domains of patient-centred care. [file 12913_2020_5082_MOESM1_ESM.docx]

**Additional File 1. Themes and exemplar quotes for strategies to tailor patient-centred care for women**

Foster patient-physician relationship

| Themes | Strategies and exemplar quotes |
| --- | --- |
| Build rapport | *Brief, friendly conversation*  First I introduce myself to them, shake their hand, make them feel that there is not any rush to do anything; that I’m not in any rush to get out. I try to find something that is common or something that’s light and try to initially make it a little bit lighter so to make them relaxed (05 male, cardiovascular disease, urban)  I ask them how they’re doing…if I haven’t met them before I introduce myself and just tell them a little bit about being a nurse practitioner and what that means. And then I ask them you know just some questions about how their life is going. I try and make it as light as possible. (26 female, contraception, urban)  *Interest in patient background*  I love to ask people things like you know, tell me something about yourself that you think is important or that you think I need to know…It could be about their work or their family life or whatever. So it’s a very open-ended question that I think gives them the opportunity to sort of you know to tell me something important about themselves (21 male, contraception, urban)  I try to get to know them as a person and get to know them where they fit in with their family and the community we have; like our appointment usually involves some like chat outside of why they’re in to see me. I try to establish a good rapport where they feel like they’re able to ask me anything (22 female, depression, rural) |
| Trusting or therapeutic relationship | *Listen to patient*  I think the way it benefits the patient is they feel that they’re being listened too and they’re not just patient number one, patient number two, but Mrs. A or Mrs. B or Mr. C (12 female, cardiovascular disease, urban)  in my experience, if a patient feels like they’re really being heard and their narrative has been understood, their hopes have been understood even if we can’t achieve them, at the very least there can be come healing which can happen…help their mental health to improve at least a little bit (34 female, depression, rural)  *Honest, open and non-judgmental tone*  I also do my best to be open and understanding of difficult circumstances that my patients may be in and be non-judgmental in my responses as well as trying to be of assistance if they’re ready to make some changes (13 female, depression, urban)  asking the question in a non-threatening matter. So sort of not, do you do drugs but like do you smoke? And if you don’t smoke cigarettes, do you smoke anything else? You know try to ask questions about lifestyle or do you have sex with men? Or sex with men and women or whatever? You know somewhat non-threatening; as if it does not matter what you say, I’m okay with it. I try to you know introduce it in a way that shows it’s something I’m comfortable with. (03 female, depression, rural)  *Body language*  I think you adopt an easy posture. I have always been a big believer in trying to remain as relaxed as possible and actually turning my back to the screen if I’m having a conversation with someone; so that I’m not just typing, it may mean that I’m a little slow in charting but it usually leads to better conversations (26 female, contraception, urban)  I make sure my body language or the words or phrases that I use are more supportive rather than just following a formula or just following some sort of questionnaire or a screening or tests. That puts them at ease and they open up quicker and it is more of a two-ended or a two-sided communication (25 female, depression, rural) |
| Comfortable physical environment | *Comfortable physical environment*  I do so first I would say in my office by the way I’ve set up my furniture, by the colors in the room, by the art on the walls. I try to create and people often comment on it, they’ll walk in and they’ll say, oh this is a, this is a cozy space. There’s like blankets and there’s a grounding bed and there’s mints (30 female, depression, urban)  I would trace it way back to just allowing people to feel safe and comfortable in a space. I have my chairs in a way that people can see the door so that their back is not to the door which is important in this work when people have mental health struggles and they may have responses like around depression where they feel like they need to be able to see the exit or need to be able to feel safe. (30 female, depression, urban)  My office is in a space – there’s green space outside, it’s an old school that’s been converted into offices so it’s not in a hospital setting which can be intimidating for a lot of people, whether man or woman…I have, you know, plants and things that are more welcoming for people in general and, you know, a small table between myself and a patient, not a large desk or anything like that (34 female, depression, rural) |

Exchange information

| Themes | Exemplar quotes |
| --- | --- |
| Understand needs, priorities and goals | Putting aside our agenda to some extent, of health care providers and, really trying to get a sense of what is important for the person sitting in front of you…it means, asking people what their goals are (32 female, contraception, urban)  Patient-centred care in my view is basically completing an individualized assessment of the patient from their perspective. So everybody obviously comes from different backgrounds and different expectations and identifying the priority for that person and then working with them to have their priorities included in the care plan that you provide for them given their medical condition (10 female, cardiovascular disease, urban)  A lot of times after I’ve talked about the diagnosis and stuff I say, now where do you see yourself? What are your expectations of this visit? Are you looking for medication? We can do counselling. We could do you know other things, social worker, that kind of stuff. So I usually sort of extend it that way to get, because often times what my impression of what the treatment should be is different than the patients impression. So I always try to ask sort of an open-ended question as to what are your expectations because some people just come because they want to talk to somebody. Some people come because they just want medication. Or some people come because there’s another whole issue like I don’t want to work and could you, if you take me off work I’ll be fine (03 female, depression, rural)  speak to them, let them tell me what their goals are, what they think are manageable and how they might want to approach that and then try to help provide them with resources to be able to do that…I think it’s important to start off with finding out what the stresses are in their life; what are the barriers to accessing care. I think you have to have an understanding of that before you move forward with trying to help the person with anything (20 female, cardiovascular disease, urban) |
| Recognize and address contextual factors | we know that any condition like depression is situated in their social context. So we definitely inquire about their social context relationship, family life, work life and any other aspect of their experience. So we want to understand the symptoms but in the context of how it operates in their end, their life…So I ask them…what’s going on with work?...what’s the situation with relationship…friendship? Are you, you know with your social interactions, family, relationship with your family of origin? So I try to get their full social context; their daily routine for example. I’ll ask them…give me a picture of your typical day (16 female, depression, urban)  we are aware of you know social determinates of health that span across different age ranges; likewise older women are more likely to be widowed and potentially be living alone and ensuring that the supports are there for them to manage their condition (27 female, cardiovascular disease, urban)  it’s really sort of finding out what the person, what their needs are and how they sort of like to approach the issue…the way that people want to approach the issue or deal with it will differ depending on their age and at what stage in their reproductive life they’re at, and those kinds of things. So I think the principals the same but the things that you may discuss or consider you know can differ with age or with you know personal preferences or even sometimes you know religious beliefs for example (21 male, contraception, urban)  *Gender*  the likelihood of intimate partner violence or, history of, you know, a power differential being experienced by a woman, would be higher. And, so, keeping that in mind, I think, changes the way that you speak to women sometimes (31 female, contraception, urban)  we should be really thinking about who the person is that we are serving and, what their experiences have been. So, women, in particular…are more likely to have experienced certain types of things…for example, I think, it's, you know, more common, particularly when thinking about things like contraception or, sexual health areas, you know, a lot of women had experienced sexual assault, or, had experienced sexual misconduct in some way (32 female, contraception, urban) |
| Assess and facilitate understanding | *Assess current understanding*  So I often start off by asking them; what is it that you understand about your illness? What is it that you understand about your treatment? (24 female, cardiovascular disease, urban)  because I teach, one of the things that I tell the residents is that one of our roles is to empower our patients to look after their own health. And for almost any problem that people come in with, the best way to do that is to you know find out what they know, find out what their understanding is; if there are gaps try and help fill those gaps by providing them with information and educational material and so on (21 male, contraception, urban)  *Use visual aids*  I have certain visual aids that I use for example; in women that are postpartum, I’ll have a chart that sort of shows baby blues which is much more common versus postpartum depression to help them sort of see visually the difference as well (19 female, depression, urban)  I often write down options for people. I talk about you know what happens in depression and the level of serotonin in their brain. I draw them a picture and then I explain that these medications raise the level of serotonin in their brain and that kind of stuff (03 female, depression, rural)  because right now we are in the media times, so I use it quite well. I actually show them a few YouTube videos which are standardized or evidence-based (25 female, depression, rural)  We have teaching kits we call them. So it’s basically a kit comprised of different types of birth control as well as tools used for like pap tests and other STI testing so that they can visually see different types of birth control, see how they would be used and going through that together (29 female, contraception, urban)  *Facilitate recall*  if it’s very complex information then I will often ask them to in their own words say it back to me so that I can make sure that they have a grasp on what the issue is… and make sure that any questions are addressed (27 female, cardiovascular disease, urban)  I’ll usually ask them you know what’s, just directly you know what’s your understanding of what we’ve been talking about or you know what’s your understanding of this condition and see you know to what extent it overlaps with you know what we’ve been talking about (21 male, contraception, urban)  Well I ask them to tell me what they’ve learned essentially (10 female, depression, urban)  *Communicate using lay language*  I try to use language that they understand as opposed to medical terminology (03 female, depression, rural)  I’m talking to them in a language and a level that they understand (27 female, cardiovascular disease, urban) |
| Provide resources | I give them the links for on-line mental health resources. They also use mental health hand-outs and also information sheets (25 female, depression, rural)  We also have reading materials, like resources such as, pamphlets or fact sheets available to give to women if they’re interested… We also sometimes use the internet going on different websites to show different resources that women can use or different apps on their phone for keeping track of when to take their birth control, things like that (29 female, contraception, urban)  I almost always provide people with educational material, patient hand-outs, that kind of stuff. In our EMR we now have a lot of that kind of material that’s embedded in the system. So we can just print things that we have decided as a group you know are well done and valuable (21 male, contraception, urban) |

Recognize and respond to emotions

| Themes | Exemplar quotes |
| --- | --- |
| Encourage expression of emotions | if I sense that they don’t feel comfortable with something, then I say, it doesn’t look like you’re comfortable with this; then why don’t we talk about that a little bit… I ask them if they have any concerns. I ask them if they have any questions. I ask them if there’s anything that worries them about any of these particular things (28 female, contraception, rural)  If I see display of emotion from something I said, and they may not have verbally expressed their reaction but they, I see there’s a change in their emotion. I will certainly prompt that you know if I notice when I say something particularly start to even become a little more withdrawn or perhaps even slightly tear up or but or if I notice any visible change in emotion I will sort of stop, stop my …we’ll stop full questions and sort of say you know what’s happened there? What’s going through your mind? And probe what the patient is experiencing in the moment (16 female, depression, urban) |
| Validate emotions | I also try to just like validate if they are expressing strong emotions which usually they do; I try to validate and normalize that too. Tell them what they’re feeling is normal or common….always a fear of judgment or stigma and a fear, or worried that they’re at fault for some reason or that it’s some sort of like inherent problem with them as a person. So we try to, I try to like validate that as that comes up but then talk about how it’s not something they should take personally about how depression is a clinical disease and try to help them see that it’s something they’re going through but not attributable to their character or anything they did (22 female, depression, rural)  reassuring them that it’s reasonable to feel the way they’re feeling and also that they’re not alone in feeling the way they’re feeling…I hear that you’re suffering and we’re gonna do what we can do to get this better (01 male, depression, rural)  I tell them all that it’s normal to be nervous and even if they’re not nervous I tell them sometimes you’re gonna have emotional ups and downs after the operation and tell them that it’s normal…I acknowledge it and I acknowledge it even if they’re not showing any emotions. That if it happens to them, it’s normal, a normal phase of the whole process. Again, it’s no different from a man or a woman (05 male, cardiovascular disease, urban) |
| Provide resources to manage emotions | They actually are connected with people with the same kind of problems so that they don’t feel alienated and alone in their struggle (18 female, depression, urban)  We also have group therapies as well, and so within my hand-out that I give everybody include a list of all the groups we offer and the contact information for who’s coordinating the groups so they can self-refer if they are interested (19 female, depression, urban) |

Manage uncertainty

| Themes | Exemplar quotes |
| --- | --- |
| Recognize uncertainty | how do we alleviate their fears and concerns, we have a conversation, a dialogue…we need to address their needs and often their social needs in dealing with where they are in their life with their health issues as well (35 female, cardiovascular disease, urban)  Often times I say, what have you heard about these things? Have you ever been on them before? Do you know anybody that’s been on them? And often times they do. And just sort of, I don’t know open-ended questions, sort of feeling out what their own concerns and biases are regarding this diagnosis and the treatment of it (03 female, depression, rural) |
| Educational or informational resources | we have developed a number of guides for all kinds of sub-populations whether they be for complex ablations, post-MI, post-surgery, transplant, heart failure. We have our own rehab guide. So we update those annually to give current information and in the past there would be you know kind of one or two guides for all. Now we have, now we’re getting very disease-specific with these guides. And we have a well-developed website as well for them (11 female, cardiovascular disease, urban)  I give them, if needed any reading materials or pamphlets…or medication side effects; that is provided to them in a written form. Or verbally too…they may meet with the pharmacist and talk about risks, benefits of treatment (15 female, depression, urban) |

Make decisions

| Themes | Exemplar quotes |
| --- | --- |
| Collaborate with patients to deliberate and make decisions | We have to ask our female patients what best serves them (11 female, cardiovascular disease, urban)  Unfortunately, my experience in medicine has been that very frequently we don’t provide as good patient-centred care as I think would be beneficial. It tends to be very focused on algorithms and what we believe is going to work, and sometimes forgetting what it is that the patient actually wants and not even asking them sometimes (34 female, depression, rural)  it’s not simply the system or who represents the systems such as the clinician or the physician that gets to define how they receive care. But also the kind of care they want. The kind of care delivery models that they’re interested in their goals. So looking at more of collaboration around patient’s kind of guiding and dictating their care within what we offer and provide (30 female, depression, urban) |
| Provide recommendations, but patients have choice | I lay out all the options and then we talk about what it is that they’re actually interested in, because a number of patients aren’t interested in medication, which is fine as long as there are other areas that they’re, you know, interested in pursing...I’m very much engaging them, engaging them with what they want and I would never obligate somebody to take something (34 female, depression, rural)  most of them I’ll just tell them like look, this is what I think we should do. This would be what I would suggest. You know will you take the pill? Will you go do counselling? Will you do this? If you won’t that’s completely fine. You know I’m open to working with you however you want. I just want to know so that we can get the best outcome for yah, so again, getting that buy-in (01 male, depression, rural) |
| Include family members or care partners | I give them the material but I also tell them that if any members of their family want to call and ask about certain things they’re welcome to call me too or bring the relatives at the next visit (12 female, cardiovascular disease, urban)  I also offer to have their partner come in for an appointment, that way I can give psycho education to the partner about their condition and the prognosis and the ways in which they can be helpful (19 female, depression, urban) |
| Improves patient compliance and outcomes | I think you would get good compliance. So if you put somebody on a drug that they really don’t want they’re not gonna take it. If you send somebody to counselling that they really don’t want to go to, they either won’t go or they won’t talk when they do go. So they have to be involved, they have to be a partner in this in order to benefit from the treatment (03 female, depression, rural)  if people are happy about their experience they will probably connect better and comply with treatment better. They feel safe in their treatment settings or with treatment personnel and would lead to better holistic care and outcomes (15 female, depression, urban)  if you know medically you’re trying to achieve something it’s only going to work if the goals of your treatment align with the goals of what the patient has in mind for their lifestyle and their priorities. So I think to achieve good outcomes…you need to be aligned with the patients understanding of what they need and what their goals are…behavioural changes is so complex and what works for one patient may not work for another (10 female, cardiovascular disease, urban) |

Enable self-management

| Themes | Exemplar quotes |
| --- | --- |
| Plan collaboratively with patients | Once they’re on the unit we start to provide them with teaching about things to look out for when they’re home. We give them lots of information, lots of pamphlets and booklets. And before I discharge them, I specifically talk to them and explain to them what exactly they should be looking out for. You know if they have, if they see something, what they should do about it. Who they should call? (17 male, cardiovascular disease, urban)  throughout the course of their stay in the hospital, we try to fully provide them education and get them ready for the discharge so that they, you know should they have any problems at home they know what to do, they know who to talk too or where to go and so forth (17 male, cardiovascular disease, urban) |
| Provide resources and programs | We have lots of groups where they learn…about symptom management (15 female, depression, urban)  very significant education website that we developed with patient input and patient validation to provide a cure rated source of relevant and important information for patients. And we offer a remote patient monitoring system that we have shown you know improves self-care and that is the standard of care in our clinic… The remote patient management system uses a rural space machine learning algorithm to help guide patient management in real-time (27 female, cardiovascular, urban) |
| Flexible follow-up appointments | we’re growing home-based, computer-based and tele-rehab sites which patients tell us has enabled and facilitated their enrollment in rehab (10 female, cardiovascular disease, urban)  I do a lot of house-calls so that’s another way to try to bridge the gap between you know how the patients feeling and…they don’t have a car or they’re afraid to go out of their house, whatever. So try to see them at home if that’s necessary (06 female, depression, rural) |

Considerations for women

| Themes | Exemplar quotes |
| --- | --- |
| Intersection of gender and other contextual factors | in terms of conversation, particularly, if we're talking about speaking with very young women or, if we're talking about women, you know, from certain cultural or, religious backgrounds. Or, you know, just somebody who has had sexual trauma or, you know, negative experiences with the health care system. So, I think, for me, the approach is one where you would try to take into account, all of those pieces (32 female, contraception, urban)  I think that there’s a lot of pressures on women that men don’t necessarily have and I’m talking about some of the social pressures; you know the pressure of having a family to take care and feeling responsible for that. Always kind of putting themselves second place and I don’t think we really look at that or set things up in a way that might be, that might help women manage that better (20 female, cardiovascular disease, urban)  There is a greater amount of shame that women carry when they’re asking for help (23 female, depression, urban) |
| Recognize women’s symptoms | I think in general women aren’t taken as serious…at least it’s the perception that physicians don’t listen to women quite the same way that they do men. You often see a woman diagnosed with anxiety based on their symptoms without a lot of investigation…I know they…potentially have quite different symptoms from the classic heart symptoms and therefore people don’t take it, they themselves might not take it as seriously, right? If they don’t, if you’re, for example, if you don’t have kind of the classic chest pain that we associate with heart attack then you’re less likely to seek attention, seek medical attention (20 female, cardiovascular disease, urban)  I just talked to a female patient two weeks ago who said that she had presented three times to the family doctor with symptoms and the family physician deemed it as you know a panic attack but she pushed back and was eventually referred to cardiology and had since had a gavage. So we know that women are referred later than men for cardiac interventions (11 female, cardiovascular disease, urban) |
| Accommodate women’s safety/caretaking needs | *Privacy*  Making sure their privacy is respected is an important aspect (10 female, cardiovascular disease, urban)  *Flexible care*  We do have childcare provided. Here there’s a playroom where people can drop off their children if they need to come to an appointment. I think that’s huge in gender-based care; allowing people some flexibility…there’s flexibility in booking appointments. So patients can reach me if they need to cancel or call and I can, I have the control to book those appointments versus going through a secretary and waiting on hold and maybe not getting to your therapist or not relaying a message (30 female, depression, urban)  Providing appointment times that are convenient for the patients, making it easy to access the clinical where the healthcare is provided… In some instances, we offer transportation to and from the hospital (13 female, depression, urban) |
| Innate comfort with female clinicians | They feel comfortable with a woman as a woman physician. They can talk intimate details especially like sexual trauma which often times, very often gets unaddressed and the reason partly maybe that they have male physicians or discomfort or shame or whatever the, unease to talk about these things with a male physician (15 female, depression, urban)  A lot of women who come to my office say that they always like a woman physician better because they seem to be a little bit more sympathetic (12 female, cardiovascular disease, urban) |

Barriers

| Themes | Exemplar quotes |
| --- | --- |
| System-level | *Lack of time*  time is always a barrier and, always a challenge to being able to, in health care, to do things the way that, I think, would be the most ideal. You know, we can't call ten minute appointments, patient-centered care, they're not. You can be the nicest doctor and, you know, you can make the most eye contact you want to, but, you know, sometimes ten minutes is just not enough (32 female, contraception, urban)  time per patient. I mean if you’re under pressure to just you know basically turn out visits, I mean you’re gonna take out the stamp and you’re gonna say, you’ve got depression here, here’s your Cipralex, go see a counsellor, come back in three months. I mean you know stamp-based medicine. It’s time related. It takes time to talk to people. It takes time to talk to people who are depressed who can’t concentrate who are sad who aren’t understanding what you’re saying. time is the biggest thing (01 male, depression, rural)  *Lack of resources*  There’s no program that’s offered specifically for women or adapted for women’s needs. So that’s actually lacking at our site…we haven’t developed like a women’s only program…I don’t think we are set-up to address the issue of you know potentially different needs or requests from women. I don’t think that’s been taken into consideration here locally, unfortunately (14 female, cardiovascular disease, urban)  We do not offer any childcare resources or supports to enable people to attend the clinic here. I don’t know if that makes a difference for some women (13 female, depression, urban)  we don’t have much in the system here for counselling mental health supports, psychologist to help them go through this (16 female, depression, rural) |
| Person-level | *Difficulty responding to contextual factors*  When it comes to mental health because it’s understood very differently in certain cultures as it is compared to people born in Canada and brought in this health care system…their concerns are somewhat different than the average person I’d see in North Bay…I think that’s where I would mainly have some difficulty, with patient literacy and my kind of cultural barriers (34 female, depression, rural)  I obviously live and work in Toronto; so you know we’re an incredibly diverse city and as a result we have many different languages and many different religions with different cultures that present in clinic and being aware of that is critically important and I think that those definitely, again, we just don’t know as much as we probably need to about how they impact but I think there’s no question they impact as well (27 female, cardiovascular disease, urban) |

Strategies

| Themes | Exemplar quotes |
| --- | --- |
| Medical training or continuing professional development | We need to teach clinicians and nursing and allied health that I think, you know, this is a female issue as well and that we need to work on patient-centered care (35 female, cardiovascular disease, urban)  training clinicians around how to best engage women, training non, the non-cardiac practitioners around how to recognize symptoms and women to be receptive about women’s concerns about their cardiac health, flagging them earlier as I say if they’ve, you know if they’re hypertensive or have high lipids or have family history (11 female, cardiovascular disease, urban) |
| Multidisciplinary teams | I think it would be good to have like more centres that would be run by PAs [physician assistants] or nurse practitioners counselling the patients about contraception. That might be like one of the things…I think it would go a long way in long term…. I sometimes get my nurse if she has time to touch base with the patients and make sure that they understand their options but in general, we need more people and more caring people to just look after these women (37 female, contraception, rural)  More interdisciplinary team-based care would be great too. Like being able to work more closely with social workers especially (22 female, depression, rural) |
| Online appointment options | We also offer appointments through video-conferencing in order to address the needs of women throughout the province of Ontario, not just those who are within the catchment area of our local hospital…which is very helpful when they have newborn babies or can’t make it physically to the appointment due to work restrictions or childcare restrictions (19 female, depression, urban) |
